# Apoptotic Death of Prostate Cancer Cells by a Gonadotropin-Releasing Hormone-II Antagonist

**DOI:** 10.1371/journal.pone.0099723

**Published:** 2014-06-13

**Authors:** Sumi Park, Ji Man Han, Jun Cheon, Jong-Ik Hwang, Jae Young Seong

**Affiliations:** 1 Graduate School of Medicine, Korea University, Seoul, Republic of Korea; 2 Department of Urology, School of Medicine, Korea University, Seoul, Republic of Korea; Roswell Park Cancer Institute, United States of America

## Abstract

Gonadotropin-releasing hormone-I (GnRH-I) has attracted strong attention as a hormonal therapeutic tool, particularly for androgen-dependent prostate cancer patients. However, the androgen-independency of the cancer in advanced stages has spurred researchers to look for new medical treatments. In previous reports, we developed the GnRH-II antagonist Trp-1 to inhibit proliferation and stimulate the autophagic death of various prostate cancer cells, including androgen-independent cells. We further screened many GnRH-II antagonists to identify molecules with higher efficiency. Here, we investigated the effect of SN09-2 on the growth of PC3 prostate cancer cells. SN09-2 reduced the growth of prostate cancer cells but had no effect on cells derived from other tissues. Compared with Trp-1, SN09-2 conspicuously inhibited prostate cancer cell growth, even at low concentrations. SN09-2-induced PC3 cell growth inhibition was associated with decreased membrane potential in mitochondria where the antagonist was accumulated, and increased mitochondrial and cytosolic reactive oxygen species. SN09-2 induced lactate dehydrogenase release into the media and annexin V-staining on the PC3 cell surface, suggesting that the antagonist stimulated prostate cancer cell death by activating apoptotic signaling pathways. Furthermore, cytochrome c release from mitochondria to the cytosol and caspase-3 activation occurred in a concentration- and time-dependent manner. SN09-2 also inhibited the growth of PC3 cells xenotransplanted into nude mice. These results demonstrate that SN09-2 directly induces mitochondrial dysfunction and the consequent ROS generation, leading to not only growth inhibition but also apoptosis of prostate cancer cells.

## Introduction

Prostate cancer is the most common malignancy that occurs in the male reproductive system. Although most prostate cancers are slow-growing, they may cause pain and difficulty in urination, and the more aggressive ones are likely to metastasize to other parts of body [1]. Globally, prostate cancer is the sixth leading cause of cancer-related death in men [2], and in the United States, it is ranked second [3]. A common treatment for advanced prostate cancer is hormonal therapy combined with radiation therapy [4]. The main goal of hormonal therapy is to remove or decrease serum androgen, a potential growth stimulant for prostate cancer. However, in many cases, the initial regression of the tumors is followed by re-growth independent of androgen levels, increased aggressiveness, and high metastatic activity [5]. For this reason, the development of effective drugs for the treatment of androgen-independent prostate cancer is an urgent issue.

In the hypothalamic-pituitary-gonadal axis, gonadotropin-releasing hormone-I (GnRH-I) synthesized in the hypothalamus stimulates the secretion of the pituitary gonadotropins luteinizing hormone (LH) and follicle-stimulating hormone (FSH), which in turn modulate the synthesis and secretion of androgens, including testosterone, from the testis [6]. Chronic administration of a GnRH-I agonist led to the down-regulation of the GnRH receptor in the pituitary gland, resulting in a marked reduction in circulating androgen levels [7]. GnRH-I antagonists also reduced serum androgen levels by inactivating the GnRH receptor [6], [8]. These results suggest that hormonal therapies using GnRH-I agonists and antagonists are applicable to the treatment of benign prostate hyperplasia and androgen-dependent prostate cancers. Furthermore, recent studies have demonstrated that GnRH-I directly affects both androgen-dependent and androgen-independent prostate cancer cells. GnRH-I agonists inhibited epidermal growth factor- or insulin growth factor-stimulated prostate cancer cell proliferation, and induced the apoptosis of the cancer cells in conditions of serum deprivation [9], [10]. These effects were suggested to be mediated by the GnRH-I receptor, which stimulates G_i_-linked signaling-dependent activation of apoptosis-related proteins, including c-Jun NH_2_-terminal kinase (JNK) [11].

In most vertebrates, the other type of GnRH, called GnRH-II, is identified, which is structurally conserved in evolution from fish to mammals [12]–[14]. GnRH-II is expressed not only in the brain but also in peripheral reproductive and immune tissues [15]. This wide expression pattern may confer a variety of physiological functions on the peptide. Similar to GnRH-I, GnRH-II is able to regulate reproduction in females by stimulating the secretion of LH and FSH [16], [17]. Even though both GnRHs act on human granulosa-luteal cells, they exhibit different hormonal regulation patterns [18], [19]. GnRH-II produced by human T cells stimulates laminin receptor expression and cell migration [20]. Interestingly, GnRH-II-induced laminin receptor expression is not blocked by the GnRH-I antagonist cetrorelix, implying that GnRH-II does not interact with the GnRH-I receptor [20]. Recently, we and other groups identified the GnRH-II receptor in non-mammalian species. The receptor binds to GnRH-II with higher sensitivity and affinity than to GnRH-I [21], [22]. Furthermore, a GnRH-II-specific receptor was cloned from monkey and is termed mammalian GnRH-II receptor [23]. The receptor is highly selective for GnRH-II and appears to be different from the GnRH-I receptor in terms of rapid internalization upon ligand interaction and signaling pathways. In human, GnRH-II receptor-like genes are localized in chromosomes 1 and 14. Although mRNAs for these genes are expressed in many tissues including the brain and even in many cell lines, they seem to be nonfunctional pseudogenes due to a premature stop codon [24], [25]. The absence of a functional G protein-coupled receptor for GnRH-II in human indicates the possibility of other types of binding partners on plasma membrane, while its functional mediators remain still unknown.

Interestingly, GnRH-II exhibits the ability to inhibit the proliferation of ovarian cancer cells as well as prostate cancer cells [26], [27]. GnRH-II is likely to function on prostate cancer cells by interacting with unknown proteins, based on our previous observation that radiolabeled GnRH-II was able to bind various human prostate cancer cells [27]. This binding was displaced by unlabeled GnRH-II, but not by GnRH-I. This finding implies that GnRH-II may be a potential drug to treat prostate cancers, even though high affinity binding of GnRH-II to prostate cancer cells appears to be not due to the expression of a conventional GnRH-II receptor. Recently, we developed a novel GnRH-II antagonist, called trptorelix-1 (Trp-1). Trp-1 is able to induce the death of androgen-dependent and -independent prostate cancer cells by underlying mechanisms such as mitochondrial dysfunction followed by reactive oxygen species (ROS) and autophagy [28].

We synthesized additional GnRH-II antagonists to compare with the Trp-1 effect and improve the death effect. Here, we present another antagonist, SN09-2 that is able to inhibit proliferation and induce death in human prostate cancer cells with a higher efficiency than that of Trp-1. Moreover, the functional mechanisms of the death are different form Trp-1, and involve the activation of apoptotic pathways.

## Materials and Methods

### GnRH-II analogues and reagents

The GnRH-II antagonist [Ac-D-Nal(2)-D-phe(4Cl)-D-Pal(3)-Ser-Phe-D-Lys-Trp-Tyr-Arg-D-Ala-NH_2_], designated as SN09-2, and its fluorescein isothiocyanate (FITC)-conjugated form were synthesized by AnyGen (Gawngju, Korea). Trp-1 [Ac-D-Nal(2)-D-Phe(4Cl)-D-Pal(3)-Ser-Tyr-D-Cit-Trp-Tyr-Pro-D-Ala-NH_2_] was also synthesized by the same company [28].

The mitochondrial membrane potential detector 5,5′,6,6′-tetrachloro-1,1′,3,3′-tetraethylbenzimidazolylcarbocyanine iodide (JC-1), MitoTracker, and 2′,7′-dichlorfluorescein-diacetate (DCF-DA) were purchased from Invitrogen (Palo Alto, CA, USA). Cell culture media, including Dulbecco's modified Eagle medium (DMEM) and Roswell Park Memorial Institute (RPMI) were obtained from Welgene Inc. (Daegu, Korea). Fetal bovine serum (FBS) and penicillin/streptomycin were from Invitrogen (Carlsbad, CA, USA). All reagents including Hoechst 33342, Annexin-V, H_2_O_2_, and bovine serum albumin were obtained from Sigma (St. Louis, MO) unless otherwise specified. Antibodies against cleaved caspase-3, intact caspase-3, and cytochrome C were purchased from Cell Signaling Technology (Danver, MA, USA). Anti-β-actin antibodies were from Sigma.

### Cell culture

All cell lines were obtained from the American Type Culture Collection (Manassas, VA, USA). PC3, LNCaP, DU145, and DLD1 cell lines were cultured in RPMI 1640 containing 10% heat-inactivated FBS, 100 units penicillin, and 100 µg/ml streptomycin at 37°C and humidified 5% CO_2_. HeLa cells were cultured in DMEM containing 10% FBS under the same conditions.

### Reporter gene assay

CV-1 Cells were culture overnight in 24-well plates and subsequently transfected with liposome complex containing pGL3/SRE-luc plasmids with bull frog GnRHR-II (bfGnRHR-II) or green monkey GnRHR-II (gmGnRHR-II). Another 48 h later, cells treated with GnRH-II (1 nM for bfGnRHR-II, 10 nM gmGnRHR-II) and different concentration of SN09-2 or Trp-1 for 6 h were washed with PBS and solublized with lysis buffer. Luciferase activity of cell extracts was determined using the standard luciferase assay system from BioTek Instrument, Inc. (Winooski, VT). To determine transfection efficiency, luciferase activities were normalized to the β-gal activity. All data are calculated from at least three independent experiments normalized to untreated groups.

### Cell viability assay

Cells were seeded in 12-well plates in triplicate (1×10^4^ cells/well). After 24 h, cells were treated with the indicated concentrations of GnRH-II antagonists solubilized in 0.1% DMSO every 24 h for the indicated number of days. Then, cells were washed with phosphate-buffered saline (PBS), dissociated with trypsin/EDTA, and re-suspended in 500 µl complete growth media. Trypan blue (0.4%) dye-excluding cells were counted under an inverted microscope (Olympus, Tokyo, Japan).

### Lactate dehydrogenase release assay

Lactate dehydrogenase (LDH) activity assay was performed according to the manufacturer's instructions (Roche Applied Science, Mannheim, Germany). In brief, PC3 cells were seeded in 12-well plates at 2×10^4^ cells/well. Next day, cells were treated with different concentrations of SN09-2 in 5% FBS-containing RPMI 1640 for 3 days. The culture media were transferred to Eppendorf tubes and centrifuged for 1 min at 5000 rpm. One hundred microliters of each supernatant was transferred into an optically clear 96-well plate. One hundred microliters of freshly prepared reaction mixture were added to each well and incubated for 30 min at 25°C. The absorbance of the samples was measured using the 490-nm wavelength filter of the ELISA reader.

### Annexin V staining assay

Apoptosis of PC3 cells was assessed using an apoptosis detection kit (Koma Biotech, Seoul, Korea). After exposure to SN09-2 for the indicated times or at different concentrations, PC3 cells were harvested and washed with cold PBS and re-suspended in binding buffer containing fluorescein isothiocyanate (FITC)-conjugated annexin V protein and propidium iodide. Annexin V binding and PI staining were determined by flow cytometric analysis (Becton Dickinson, San Jose, CA, USA). Apoptotic cells were defined as PI-negative and annexin V-positive.

After treatment with SN09-2, PC3 cells on poly-D-lysine-coated cover slips were fixed with 4% paraformaldehyde for 15 min, washed with PBS three times, and incubated with Cy3-conjugated annexin V. Cells were washed with PBS and shortly incubated with Heochst33342 (10 µg/ml). Stained cells were visualized under an LSM510 confocal microscope (Carl Zeiss, Jena, Germany).

### Western blot analysis

Cells (5×10^4^) were plated in 100-mm dishes. The next day, cells were treated with the indicated concentrations of SN09-2 for different times, harvested, and incubated with buffer containing 10 mM HEPES (pH7.9), 10 mM KCl, 0.5 mM DTT, and protease inhibitor cocktail (Roche) for 30 min on ice. Cells were disrupted with a Dounce homogenizer by 20 strokes and centrifuged at 5,900×*g* for 10 min to collect the cytosolic fraction. The resulting pellets were re-suspended in cold buffer, briefly sonicated three times, and centrifuged at 5,900×*g* for 10 min to obtain crude mitochondrial proteins. Proteins of each fraction (20 µg) were applied to SDS-polyacrylamide gel electrophoresis (12% gel) and transferred to polyvinylidene difluoride membranes (Millipore, Billerica, MA). The membranes were blocked with 3% bovine serum albumin and incubated with the appropriate antibodies. After washing with Tris-buffered saline containing 0.05% Tween 20, membranes were incubated with horseradish peroxidase-conjugated secondary antibodies and the signals were detected using an enhanced chemiluminescence solution (GE Healthcare Life Science, Buckinghamshire, UK).

### Subcellular localization of SN09-2

Two days after plating on poly-D-lysine-coated cover slips in 12-well plates, PC3 cells were treated with 66 nM MitoTracker for 30 min and then with 10 mM FITC-SN09-2 for 10 min at 37°C. After washing with PBS, the cells were fixed with 4% paraformaldehyde for 10 min. Nuclei were stained with Hoechst 33342. Fluorescence signals were detected under the confocal microscope.

### Measurement of mitochondrial membrane potential

Mitochondrial membrane potential was measured using JC-1. In healthy cells, JC-1 forms aggregates within mitochondria, generating red fluorescence. However, during mitochondrial depolarization, JC-1 monomers diffuse throughout the cell, producing green fluorescence. PC3 cells were seeded on poly-D-lysine-coated cover slips in 12-well plates. After treatment with 10 µM SN09-2 for 3 days, cells were incubated with PBS containing JC-1 (2.5 µg/ml) for 20 min at room temperature. Cells were washed, fixed with 4% paraformaldehyde, and observed under the confocal microscope. After incubation with JC-1, PC3 cells were detached from dish and applied for fluorescence-activated cell sorting (FACS) analysis.

### Beads conjugation and pull-down assay

Beads conjugation of SN09-2 was performed as described in previous paper [28]. In brief, N-hydroxysunninimide-activated sepharose beads were incubated with SN09-2 in coupling buffer overnight at 4°C, and then washed with a buffer containing 0.1 M Tris-HCl (pH 8.5) and a buffer containing 0.1 M acetate (pH 4.5) and 0.5 M NaCl. Membrane fraction of PC3 cells was isolated by homogenization and centrifugation. The resulting pellets were dissolved in lysis buffer containing 1% Triton-X 100 and centrifuged at 20,000×*g* for 15 min at 4°C. Supernatants were incubated with SN09-2-conjugated sepharose and washed with lysis buffer. The precipitates were incubated with 50 µM SN09-2 and the supernatants were applied for SDS-PAGE and Western blotting with anti-GPR75 antibodies.

### Intracellular ROS generation

Reactive oxygen species (ROS) were measured using DCF-DA. PC3 cells (1×10^5^) were plated on 60-mm dishes, cultured in 5% FBS RPMI, and treated with SN09-2 for 3 h. After incubation with 5 µM DCF-DA for 30 min at 37°C in the dark, cells were washed, re-suspended in PBS, and then applied to FACS analysis.

### Immunocytochemistry

PC3 cells (4×10^4^) were plated on cover slips in 12 well plates and treat with 10 µM SN09-2 for 12 h. The cells were fixed in 4% paraformaldehyde for 10 min, permeabilized with 0.2% Triton-X in PBS, washed and blocked with 3% BSA in PBS for 30 min. Polyclonal rabbit antibodies against cleaved caspase-3 (1∶200) and monoclonal mouse anti-β-actin antibodies (1∶1000) were applied to the cells. Active caspase-3 and β-actin were visualized by incubating with both Alexa 594-conjugated anti-mouse and Alexa 488-conjugated anti-rabbit secondary antibodies for 1 h. Then cells were stained with Hoechst 33342 (1 µg/ml) for 5 min, mounted onto slides, and observed under the confocal microscope.

### *In vivo* growth of prostate cancer cells

Six-week-old male athymic nude mice (BALB/c-nu) were obtained from Harlan (Houston, TX). Mice were housed under specific pathogen-free conditions in an individually ventilated caging system. All experimental procedures were performed after receiving approval from the Institutional Animal Care and Use Committee of Clinical Research Institute in Seoul National University Hospital. PC3 cells (6×10^6^) were injected into the right flank of the mice. Ten days after cell injection, tumors with volumes of around 20–25 mm^3^ developed in most mice; tumor volumes were measured every day. Tumor volumes were determined by two perpendicular dimensions [length (*L*) and width (*W*)] and the height (*H*), measured using Vernier calipers (the formula is *V* =  (π/6) × (*W*×*L*×*H*). From this time, SN09-2 (acetic acid form) dissolved in 5% N,N-dimethylacetamide (DMA), 10% glycerol, and 85% distilled water which is biocompatible solvent were subcutaneously injected into the mice for 25 consecutive days. The tumor size in each mouse was measured every day. Six to seven mice were used for each experimental group.

### Statistical analysis

Data were analyzed using PRISM4 software (GraphPad). Group means were compared using Student's *t* test or one-way ANOVA followed by Bonferroni's multiple comparison. A *P-*value <0.05 was accepted as significant.

## Results

### SN09-2 induces prostate cancer cell-specific death

**Figure 1:**
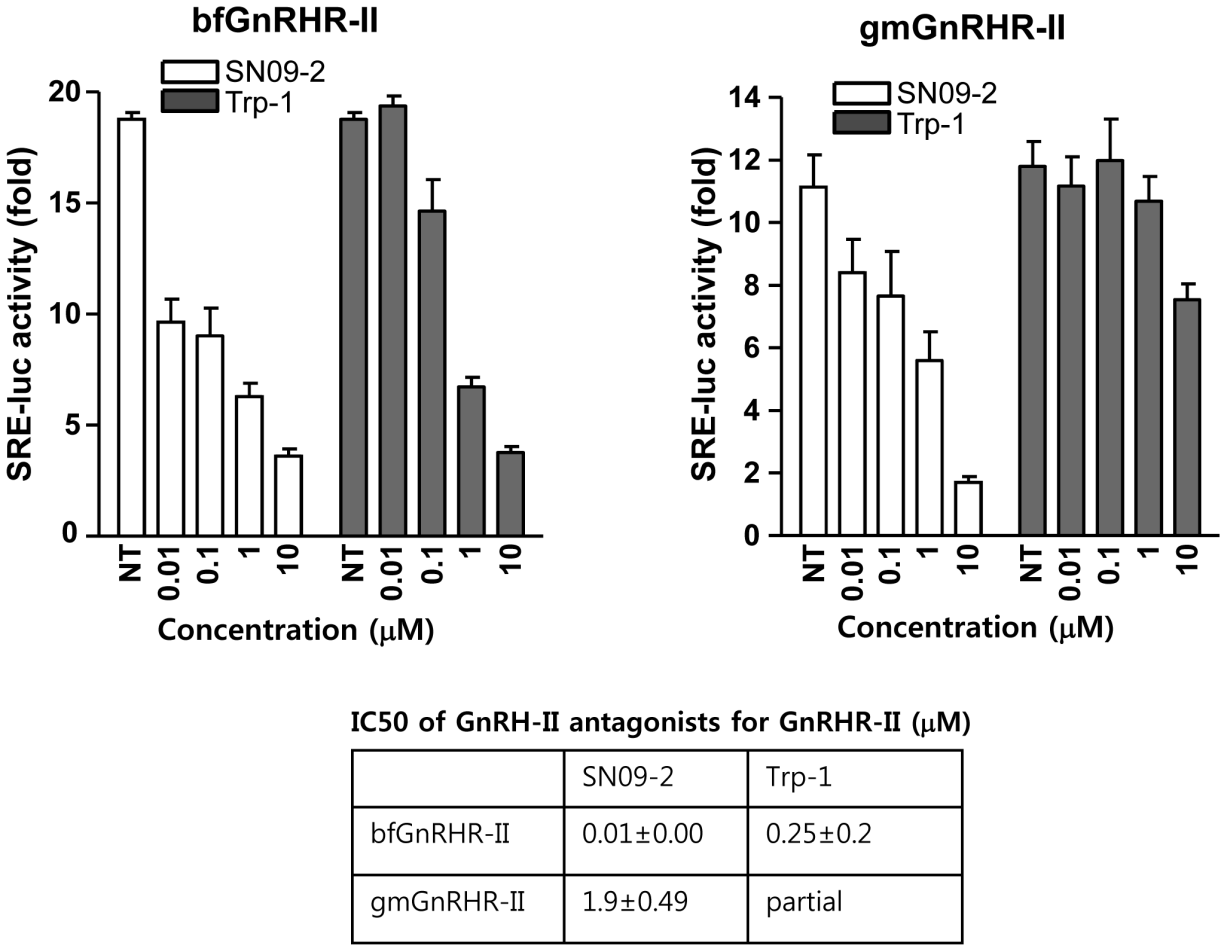
Dose-response of inhibition by GnRH-II antagonists. Either bfGnRHR-II or gmGnRHR-II was transfected with SRE-luc reporter into CV-1 cells. Cells were treated with the antagonists of different concentration in the presence of GnRH-II (1 nM for bfGnRHR-II, 10 nM gmGnRHR-II).

**Figure 2:**
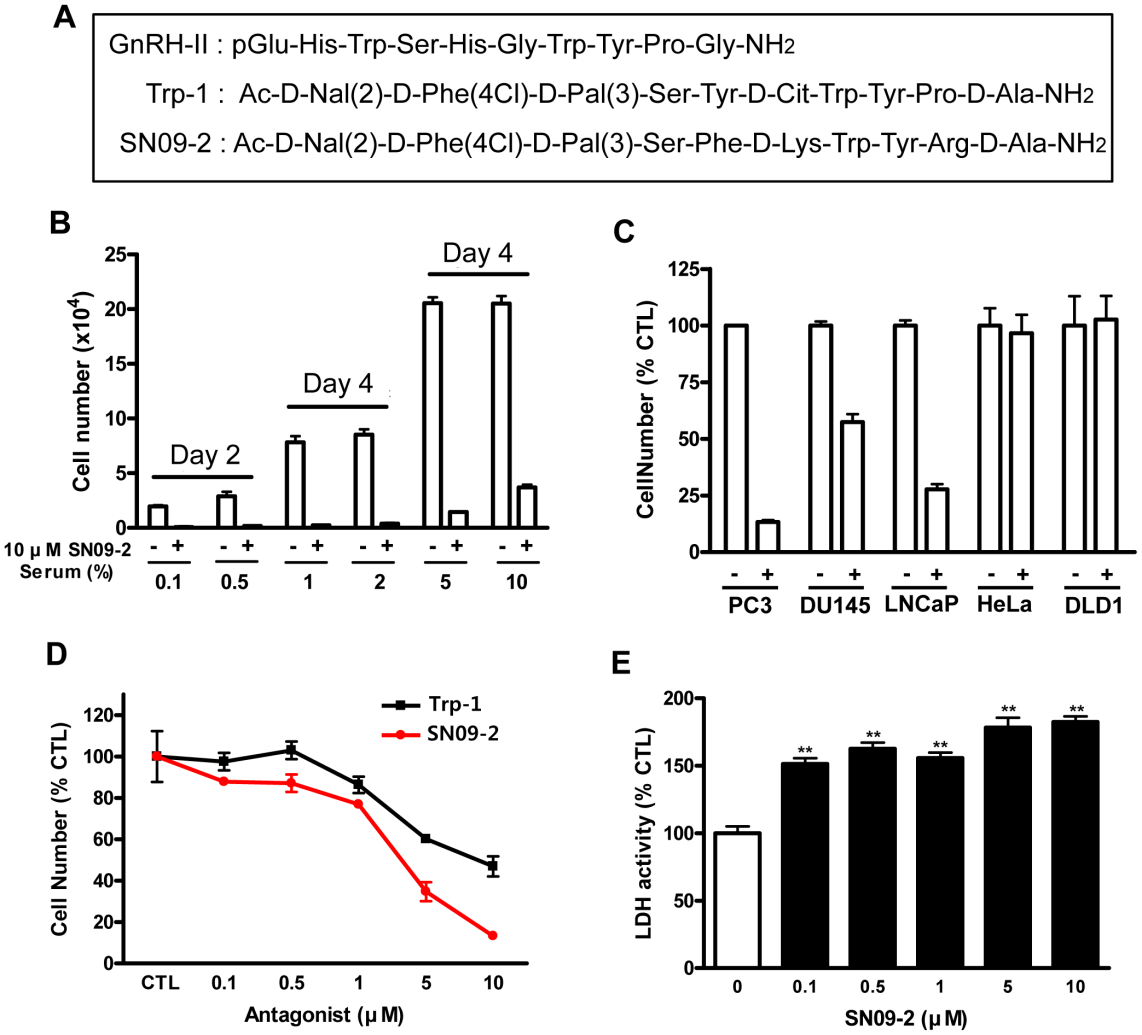
SN09-2 inhibits prostate cancer cell growth. (A) Molecular sequences of GnRH-II, Trp-1, and SN09-2 (B) PC3 cells were incubated in RPMI media containing various concentrations of FBS and exposed to 10 µM SN09-2 for 4 days. The number of viable cells was counted under a light microscope. (C) PC3, Du145, LNCaP, HeLa, and DLD1 cells were incubated with 5% FBS media containing 10 mM SN09-2 or DMSO for 3 days. (D) PC3 cells were treated with different concentrations of Trp-1 or SN09-2 for 3 days, and then viable cells were counted under the light microscope. The cell number of each group was compared with the DMSO-treated group. CTL: DMSO-treated. (E) PC3 cells were treated with various concentrations of SN09-2 for 3 days. Using culture supernatants from each group, LDH activity was determined as described in Materials and Methods. Data are means ± S.E. *, *p<*0.05; **, *p<*0.01, versus control.

We previously reported that a novel GnRH-II antagonist Trp-1 induced prostate cancer cell death [28]. To identify more effective molecule, we synthesized 65 analogues based on Trp-1 structure and screened their death activities on PC3 and LNCaP without any toxic activity on lung fibroblast MRC-5. Finally, SN09-2 was chosen as potentially effective GnRH-II antagonist to inhibit prostate cancer cell growth. In previous reporter, we identified Trp-1 has antagonistic activity on GnRHR-II [28]. Our present reporter gene assay reveals that SN09-2 also has a potent antagonistic activity on GnRHR-II even compared with Trp-1 (Fig. 1). SN09-2 efficiently antagonized the effect of GnRH-II on bull frog GnRHR-II (IC_50_ =  0.01 µM) and green monkey GnRHR-II (IC_50_ =  1.9 µM). However, Trp-1 inhibited activation of only bull frog GnRHR-II with high potency (IC_50_ = 0.25 µM). The sequences of GnRH-II, Trp-1, and SN09-2 were described in Fig. 2A. PC3 cells (androgen-independent prostate cancer cells) were treated with 10 µM SN09-2 in culture media containing different concentrations of FBS. In low serum concentrations (less than 2%) SN09-2 induced more than 95% growth inhibition of PC3 cells within 4 days. And even in higher serum concentrations, SN09-2 dramatically inhibited cell growth by more than 85%, although the inhibition was slightly affected by serum concentration (Fig. 2B). Treatment with SN09-2 for 4 days induced 91% and 88% growth inhibition of PC3 cells in 5% and 10% serum conditions, respectively. This result indicates that SN09-2 negatively regulates prostate cancer cell growth in a serum-independent manner. Next, various cancer cells were treated with 10 µM SN09-2 in 5% serum-containing media to determine the prostate cancer cell-specific effect of the GnRH-II antagonist. Three days after treatment with SN09-2, the growth of PC3 and LNCaP cells was drastically downregulated, and that of DU145 cells was also inhibited by ≈50%, suggesting that sensitivity of the prostate cancer cells to SN09-2 may be various. However, the growth of cells from other tissues such as HeLa (cervical cancer) and DLD1 (colon cancer) was not affected by SN09-2 (Fig. 2C). Although more cells should be tested, these results may indicate SN09-2 has prostate cancer cell-specific growth inhibitory effect.

In a previous report, we demonstrated that Trp-1 inhibited the proliferation of PC3 cells [28]. To investigate the growth inhibition efficiency of the GnRH-II antagonists, we added them to PC3 cells cultured in 5% serum condition for 3 days. In comparison with control groups, both antagonists significantly attenuated cell growth in a dose-dependent manner. Furthermore, in all groups treated with different concentrations, cell growth was more potently inhibited by SN09-2 rather than Trp-1, suggesting that SN09-2 is likely to be better molecule for development as a prostate cancer inhibitor (Fig. 2D). The inhibitory effect on cell growth may be correlated with cell death. To confirm the idea, we measured LDH activity from cell culture media treated with different doses of SN09-2. As shown in Fig. 1E, the activity was increased in all SN09-2-treated cells. This result suggests that SN09-2 is able to stimulate prostate cancer cells to death even in small amounts. Even though a high dose of SN09-2 slightly increased LDH activity, the activity was not correlated with the dose of the agonist, implying that this assay seems to be too sensitive to distinguish dose-dependent activity on cell death.

### Mitochondrial accumulation of SN09-2

**Figure 3:**
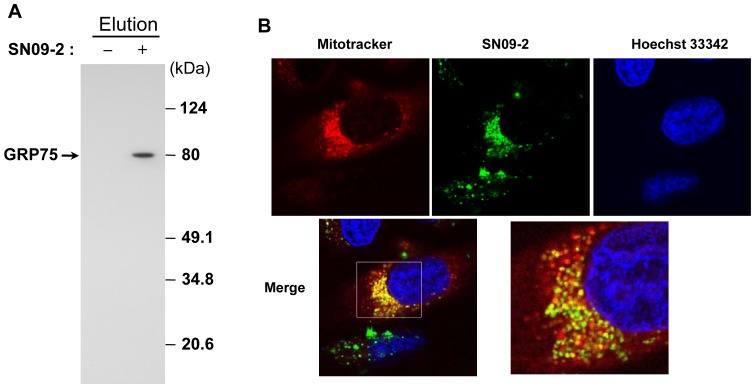
Subcellular localization of SN09-2 in PC3 cells. Cells were incubated with FITC-conjugated SN09-2 and MitoTracker. After washing, the cells were briefly labeled with Hoechst33342 (for nucleus staining). The images were taken under a confocal microscope. Red: MitoTracker, Green: FITC-SN09-2, Blue: Hoechst33342

In previous reports, we and other groups observed that radiolabeled GnRH-II bound to prostate cancer cells through interaction with an approximately 80-kDa protein whose identity was designated as glucose-regulated protein 75 (GRP75) based on liquid chromatography-electrospray ionization-tandem mass spectrometry [11], [28], [29]. Interestingly, our pull-down assay also showed that SN09-2 directly interacted with GRP75 (Fig. 3A). Since GRP75 is found predominantly in mitochondria, the final destination of SN09-2 may not be the cell surface. To explore the subcellular location of the antagonist, we labeled SN09-2 with FITC and added it to PC3 cells at a 1 µM concentration for 10 min. The FITC-SN09-2 signal was not seen on the plasma membrane but around the nucleus. When treated with MitoTracker dye, the FITC signal overlapped with that of the dye, indicating that FITC-SN09-2 can accumulate in the mitochondria (Fig. 3B). FITC-SN09-2 was hardly detectable in other cell lines such as HeLa, MCF7, and DLD1 (data not shown). Mitochondrial accumulation of this conjugate is quite similar to that of FITC-conjugated GnRH-II and Trp-1, which were attenuated by unlabeled GnRH-II. This result implies that an unknown endocytosis pathway for GnRH-II and its antagonists may exist in prostate cancer cells.

### Mitochondrial damage and ROS induction in SN09-2-treated cells

**Figure 4:**
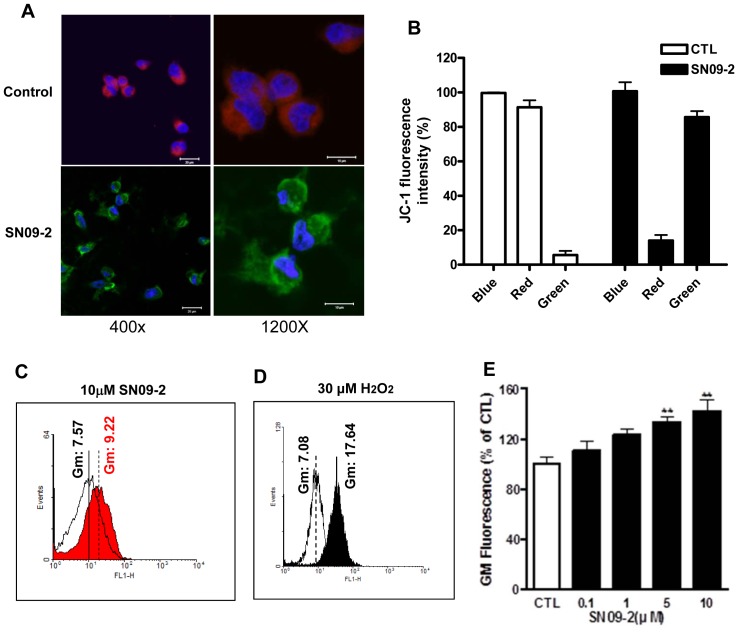
SN09-2 decreases mitochondrial membrane potential and induces ROS generation. (A) After treatment with 10 µM SN09-2 for 3 days, PC3 cells on cover slips were incubated with JC-1, fixed with 4% paraformaldehyde, briefly stained with Hoechst33342, and observed under the confocal microscope. (B) SN09-2-treated PC3 cells were detached from the dish and applied to FACS analysis to examine the fluorescence color change. (C) PC3 cells treated with SN09-2 for 3 h were incubated with DCF-DA, and applied to FACS analysis (D) Positive control of intracellular ROS. PC3 cells were treated with H_2_O_2_ for 5 min, labeled with DCF-DA, and then applied to FACS analysis. (E) Geometric means of fluorescence signals in PC3 cells treated with different concentrations of SN09-2 are shown as graphs. Data are means ± S.E. **, *p<*0.01, versus control.

Mitochondrial dysfunction is thought to be directly related to cellular death [30]. Mitochondrial targeting of SN09-2 may be linked to the death effect on prostate cancer cells through the dysfunction of the organelle. We investigated the electric potential change in the mitochondrial membrane, which indicates mitochondrial function. As shown in Fig. 4A, 3-day treatment of PC3 cells with SN09-2 decreased red fluorescence intensity around nucleus, but increased yellow and green fluorescence by more than 80% in the cytoplasm, indicating a decrease in the mitochondrial membrane potential (Fig. 4B). In many cases, mitochondrial dysfunction is a prerequisite for the generation of cytoplasmic ROS. When PC3 cells were treated with 5 µM SN09-2, cytoplasmic ROS levels, measured by the fluorescence of the fluoroprobe DCF-DA, were increased (Fig. 4C). Although the geometric mean of the fluorescence was much lower than that seen in H_2_O_2_-treated cells, the value may be enough to indicate alteration of mitochondrial function (Fig. 4D). Furthermore, SN09-2 increased cytoplasmic ROS levels in a dose-dependent manner (Fig. 4E).

### SN09-2 induces cell death through the activation of apoptotic signaling pathways

**Figure 5:**
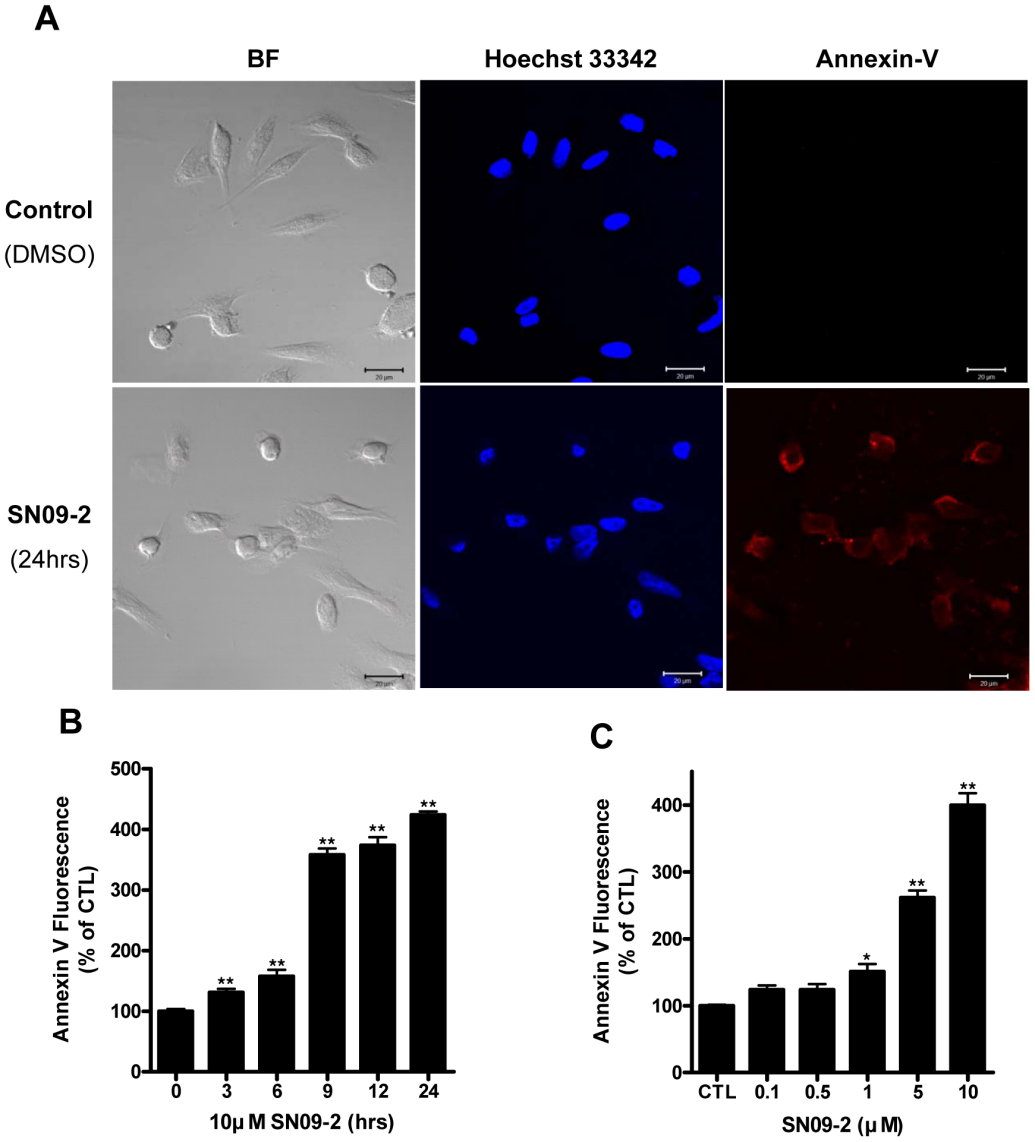
SN09-2 induces apoptosis of PC3 cells. (A) PC3 cells treated with 10 µM SN09-2 on cover slips were incubated with Cy3-conjugated annexin V, and briefly stained with Hoechst33342. Fluorescence images were taken under a fluorescence microscope. BF: cell morphology under light microscope (B, C) Annexin V-positive cells were increased in a dose- and time-dependent manner (B) PC3 cells treated with SN09-2 were harvested at different time points, incubated with FITC-conjugated annexin V, and applied to FACS analysis (C) PC3 cells were treated with different concentrations of SN09-2 for 12 h before annexin V staining. Data are means ± S.E. *, *p<*0.05; **, *p<*0.01, versus control.

Mitochondrial dysfunction and the consequent ROS generation may precede apoptosis, a type of cell death. To determine mechanisms underlying cell death stimulated by SN09-2, we stained PC3 cells with Cy3-conjugated Annexin V, which specifically binds to membrane phosphatidylserine. PC3 cells treated with 10 µM SN09-2 for 24 h were extensively stained with Cy3-Annexin V, suggesting that the cells undergo apoptotic change (Fig. 5A). When cells were applied to FACS analysis after staining with FITC-conjugated Annexin V and propidium iodide, most cells were FITC-positive but PI-negative, implying that SN09-2 stimulated apoptotic death but not necrotic death. Annexin V staining was dramatically enhanced by 9 h after 10 µM SN09-2 treatment and sustained until 24 h (Fig. 5B). Under low concentrations of SN09-2, few cells were FITC-positive, whereas most cells treated with high concentrations (more than 5 µM) were FITC-positive, suggesting that SN09-2 is an efficient apoptosis inducer for prostate cancer cells (Fig. 5C). Together with the proliferation result, the rare staining of the cells treated with less than 0.5 µM SN09-2 demonstrates that a low dose of the antagonist inhibits cell growth rather than stimulating cell death.

**Figure 6:**
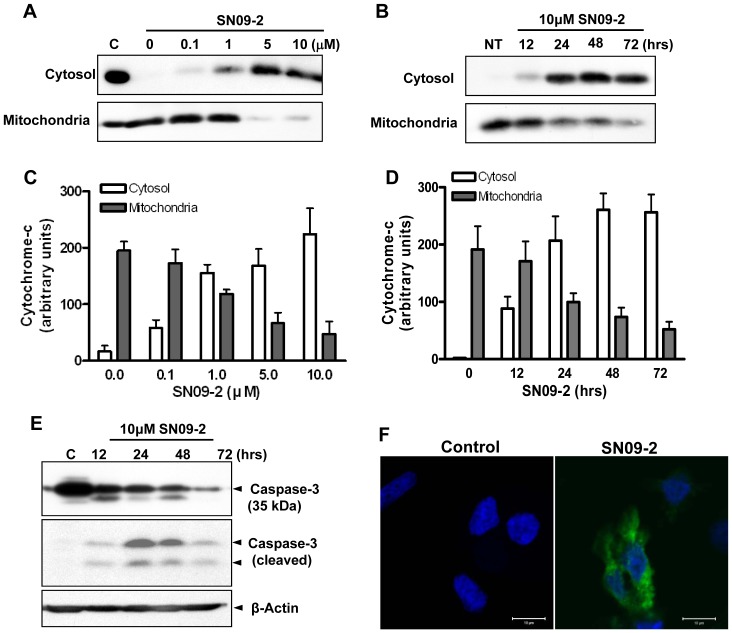
SN09-2 stimulates cytochrome c release from mitochondria and caspase-3 activation. (A–D) Western blotting of cytochrome c in the cytosolic and mitochondrial fractions (A, C) PC3 cells were treated with different concentrations of SN09-2 for 24 h and fractionated into cytosol and mitochondria. 20 µg lysates from each fraction were used for SDS-PAGE and western blotting with anti-cytochrome c antibodies. (B, D) Cells treated with 10 µM SN09-2 were harvested at different time points, and then fractionated. (C, D) The signal intensity of each blot was measured and shown as graphs (E) PC3 cells treated with 10 µM SN09-2 were harvested at different time points, and used for western blotting with anti-caspase-3 or a cleaved form of caspase-3 antibodies. The amount of loaded proteins was normalized with anti-β-actin antibodies. (F) SN09-2-treated PC3 cells on cover slips were labeled with antibodies for active cleaved caspase-3 and anti-β-actin, and then briefly stained with Hoechst33342. Fluorescence signals were observed under a confocal microscope.

The release of cytochrome c from mitochondria is an early event during caspase-dependent cell death. Upon the release of cytochrome c into the cytoplasm, it binds apoptotic protease activating factor-1 and procaspase-9 to form the apoptosome, which subsequently activates caspase-3 and -7 responsible for destroying the cells [31]. Western blot analysis revealed that SN09-2 stimulates cytochrome c release into the cytoplasm in a time- and dose-dependent manner. The most cytochrome c was released from mitochondria in cells treated with more than 5 µM SN09-2 (Fig. 6A, C), and cytosolic saturation of the protein was accomplished by 24 h after treatment (Fig. 6B, D).

Next, we examined caspase-3 activation by SN09-2. When PC3 cells were treated with 10 µM SN09-2 for different times, the intensity of intact caspase-3 gradually decreased, while the amount of cleaved caspase-3 was maximal after 24 h of treatment, and then decreased (Fig. 6E). We also investigated caspase-3 cleavage in LNCaP and DU145 cells. The cleavage patterns were quite similar to those in PC-3 cells, but the efficiency was low, which is correlated with different death or growth inhibition activity on those cells in Fig. 2C (data not shown). Immunocytochemistry for actively cleaved caspase-3 in PC3 cells revealed that a strong fluorescence signal was detected in most cells treated with SN09-2 (Fig. 6F). This result indicates that SN09-2 stimulates prostate cancer cell death by activating the apoptotic pathway. According to previous results, Trp-1 could not trigger the activation of the apoptotic machinery even though the antagonist accumulated in mitochondria and induced cell death via autophagic pathways. The different functional mechanisms of the antagonists in cell death may indicate that SN09-2 is able to induce prostate cancer cell death with higher potency by stimulating the caspase activation cascade, compared with Trp-1, which utilizes autophagic cell death.

### SN09-2 down-regulates *in vivo* prostate cancer cell growth

**Figure 7:**
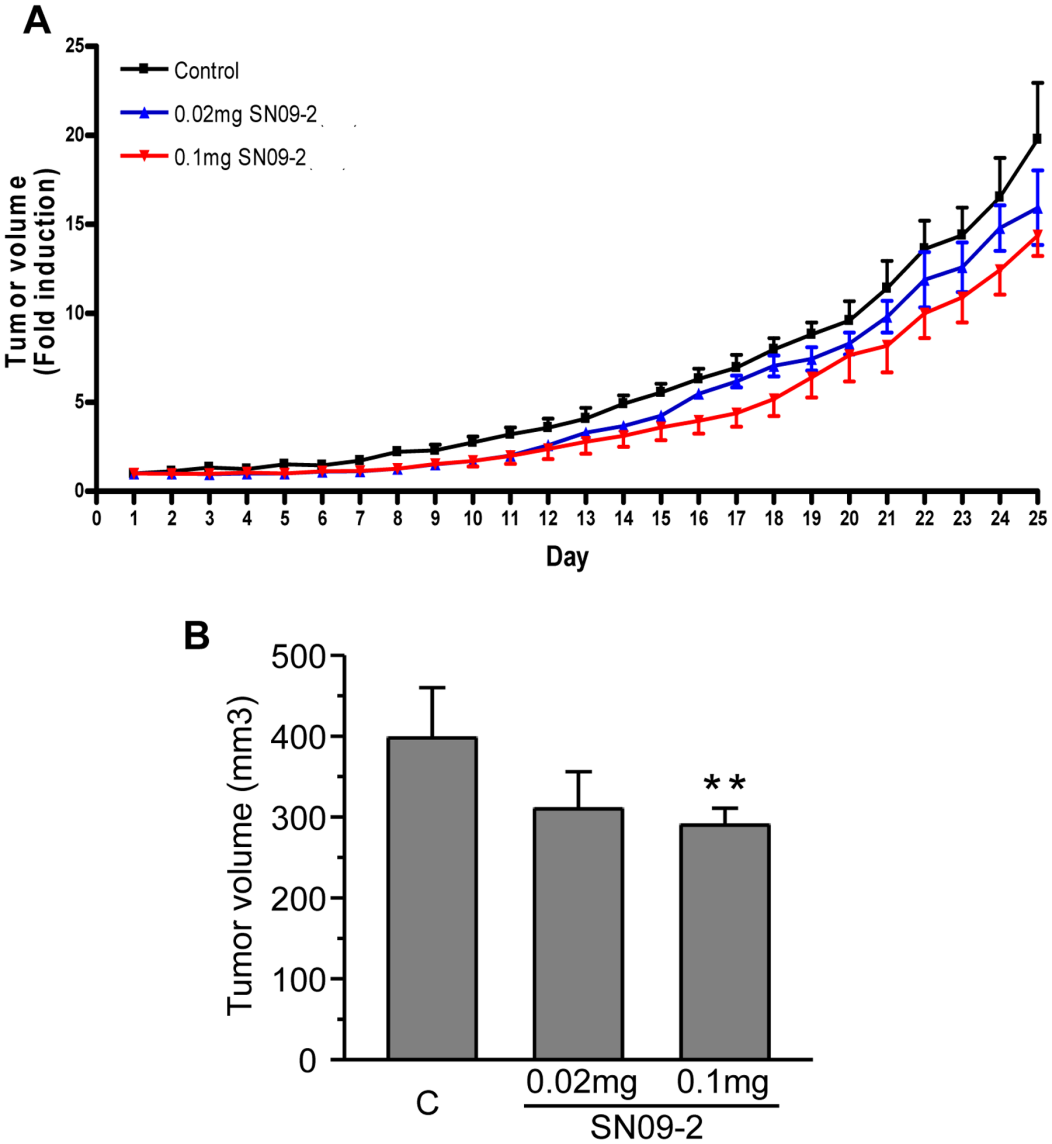
SN09-2 inhibits the growth of PC3 xenografts in nude mice. (A, B) Effect of SN09-2 on PC3 cell growth in nude mice (n = 6 for each group). PC3 cells were injected into the flanks of the mice. After 10 days, the mice were subcutaneously injected with different doses of SN09-2 for 25 consecutive days. Data represent fold increase of tumor volumes over those for the first day injection. (B) Tumor volumes of each group are presented on day 25 after SN09-2 injection. Data are means ± S.E. **, *p<*0.01, versus control. C: control.

PC3 cells were implanted into the flanks of nude mice. After tumor formation, SN09-2 was injected into the mice at different concentrations for 25 consecutive days. Treatment with 0.02 mg SN09-2 slightly decreased tumor volume with no statistical significance. In the group treated with 0.1 mg SN09-2, tumor volumes were reduced by 27%, compared with the vehicle-treated group (Fig. 7A, B). This inhibitory activity of SN09-2 was much lower than that of Trp-1 (60% at 0.1 mg), even though SN09-2 efficiently inhibited proliferation and even potently stimulated the death of PC3 cells *in vitro*. The discrepancy between *in vitro* and *in vivo* activity may be due to the solubility of the antagonist. SN09-2 was dissolved well in DMSO, a solvent for *in vitro* treatment. However, although it appeared well dissolved in 5% N,N′-dimethylacetamide (DMA), a formulation used for Trp-1 animal treatment, it remained as a solid mass at the injection site for a while, which might have led to a reduction in the amount of functional SN09-2 in the animal. After sacrificing the animals, we performed histological and blood chemistry analyses, and found that the liver and kidney of SN09-2-treated mice exhibited no histo-pathological abnormalities. In addition, the serum levels of alkaline phosphatase, aspartate aminotransferase, alanine aminotransferase, urea nitrogen, creatinine, and total bilirubin in the serum did not differ between vehicle and SN09-2-treated mice (data not shown). This result indicates that long term SN09-2 treatment has negligible toxicity on the liver and kidney. Taken together, if a suitable solvent formulation is identified for the *in vivo* usage of SN09-2, it can be developed as a useful drug for prostate cancer treatment. Some GnRH antagonists containing D-Arg^6^ or D-Lys^6^ has been reported to exhibit the edema effect by triggering histamine release. SN09-2 also has D-Lys^6^, which may compromise its utility as a clinical candidate, although edema-like phenotype was not observed in the mice. In the future study, potential effect on histamine release from mast cells should be investigated.

## Discussion

Both GnRH-I agonists and antagonists are able to inhibit the growth of cancer cells in sex hormone-regulated tissues including ovarian cancer and prostate cancer, by directly stimulating cell death pathways as well as by decreasing gonadotropin and sex hormone levels [11], [32], [33]. Because of the sequence similarity between GnRH-I and GnRH-II, GnRH-II and its antagonists are considered to exert similar effects on these types of cancer, although its receptor has not been identified in human and its action mechanisms have not been well elucidated. In some reports, GnRH-II has been shown to induce a stronger anti-proliferative effect than that of GnRH-I in ovarian cancer [33]. Our previous studies revealed that the GnRH-II antagonist Trp-1 inhibited proliferation and stimulated death signaling in human prostate cancer cells through mitochondrial dysfunction and autophagy [27], [28]. Trp-1 also inhibited growth of the tumor formed by implanted androgen-independent PC3 prostate cancer cells, whereas the GnRH-I antagonist cetrorelix had a very weak effect on tumor growth. These results suggest that GnRH-II may be a good target to develop drugs for hormonal therapy in prostate cancer. The present study shows that the novel GnRH-II antagonist SN09-2 exerts a potent inhibitory effect on PC3 cell growth *in vitro* compared with Trp-1. This anti-proliferation effect seems to be highly specific for prostate cancer, since SN09-2 does not inhibit the growth of cells originating from different tissues.

Although the growth inhibitory effect of GnRH-II antagonists in sex hormone-related cancer cells is obvious, the mechanisms underlying the GnRH-II antagonist-mediated growth inhibition and even death of these cells are not completely understood. In the case of GnRH-I, its receptor-mediated signaling pathways have been generally defined in the pituitary and are preferentially coupled to G_q/11_, resulting in the activation of phospholipase C, subsequent protein kinase C activation, and cytosolic calcium ion increase [34]. However, in cancer cells, the signaling pathways are quite complicated [35]. GnRH-I receptors are linked to G_i_ although these cells still retain G_q/11_/phospholipase C signaling machinery [27]. In serum-deprived conditions, GnRH-I agonists may activate c-Jun N-terminal kinase (JNK)-mediated signaling and concomitantly reduce phosphatidylinositol 3-kinase/Akt signaling in cancer cells [11]. Other studies reported that the activation of protein kinase C and extracellular signal-related kinase 1/2 are necessary for GnRH-induced ovarian cancer cell death [36], but it is still uncertain whether these early signaling events are mediated by the cognate receptor or nonspecifically activated by the antagonists. Although the GnRH-I receptor is a well-known mediator in canonical signaling pathways triggered by GnRH-I, the role of the receptor in the action of GnRH-I in cancer cells is controversial [33], [37]. These conflicting experimental results complicate our understanding of how GnRHs induce prostate cell death.

A GPCR-like receptor specific for GnRH-II does not exist in human; this may be the reason that the functional activity of the peptide has not been defined precisely. Although some researchers have reported that the GnRH-I receptor is able to mediate GnRH-II-stimulated cellular responses [37], [38], there is other evidence that GnRH-II has distinctive roles that cannot be explained by the GnRH-I receptor. For example, down-regulation of the GnRH-I receptor in ovarian and endometrial cancer cells abolished the growth inhibition activity of GnRH-I, but not that of the GnRH-II antagonist [33]. Although SN09-2 has antagonistic effect on GnRHR-II at subnanomolar concentration, particularly with respect to the action on prostate cancer cells, this molecule induces cell death at higher concentration (micromolar level). By this reason, it is speculated that SN09-2 may function through uncharacterized membrane proteins, but its final destination is likely to be mitochondria in a prostate cancer cell-specific manner, which is distinct from GnRH receptor-mediated endocytosis.

The cell-specific penetration mechanism of GnRH-II through the plasma membrane may generate significant attention, because it would provide a useful method for drug delivery to the inside of cell. The idea of GnRH-II binding to unknown membrane protein was supported by interaction between radiolabeled GnRH-II and prostate cancer cells which was replaced with unlabeled GnRH-II but not with GnRH-I. In addition, we identified a GnRH-II-interacting ∼80-kDa protein in human prostate cancer cells, using photoaffinity labeling [27]. Proteomic analysis following a pull-down assay with GnRH-II antagonists revealed that the protein band includes ∼80-kDa mitochondrial proteins such as GRP75, TRAP-1 and HADHA [28]. These proteins do not explain the mechanism by which GnRH-II antagonists penetrates the outer membrane of prostate cancer cells, indicating that GnRH-II antagonist -interacting membrane proteins should be further defined. This failure may be due to the possibility that interaction between GnRH-II antagonist and a protein in the plasma membrane of prostate cancer cells is too reversible or dynamic to be detected by biochemical analysis. However, along with co-localization of MitoTracker with Trp-1 or SN09-2, these molecular interactions support the idea that GnRH-II antagonists are finally destined to mitochondria [28]. Precipitation of GRP75 in the pull-down assay with SN09-2 also indicates that SN09-2 can be localized to mitochondria.

Mitochondrial proteins play pivotal roles in organelle-specific functions such as energy conversion, calcium hemeostasis, ROS generation, and electron transport [39], [40]. The disturbance of such mitochondrial functions is a critical step towards cell death through the activation of either apoptotic or autophagic pathways [41], [42]. GRP75 a mitochondrial chaperon is an essential protein for mitochondria biogenesis by refolding and sorting mitochondrial proteins. Elevation of GRP75 is easily observed in various cancer cells, while knockdown of the protein induces mitochondrial dysfunction leading to cell death [43], [44]. It is reasonable to assume that SN09-2 inhibits proliferation and even induces cell death by interacting with mitochondrial proteins, especially GRP75, and disturbing their functions. Functional assays using fluorescent dye indicate that SN09-2 significantly decreases the mitochondrial membrane potential and increases cellular ROS levels. As a result of mitochondrial dysfunction, cytochrome c is released from the mitochondria to the cytosol, and subsequently caspase-3 is activated, leading to apoptotic death of the prostate cancer cells. Interestingly, both SN09-2 and Trp-1 have the same effect on mitochondria, such as membrane potential alteration and ROS generation, although they activate distinct cell death processes. Trp-1 does not induce to cytochrome c release and caspase-3 activation, but rather induces autophagic cell death. Autophagy has been thought to act as a cellular protective mechanism by removing unnecessary or dysfunctional cellular components through the lysosomal machinery [45]. In some disease conditions, autophagy appears to promote cellular adaptation and survival in response to stress [46]. However, excess autophagy appears to promote cell death, suggesting that the extent of the autophagic process may define the destiny of the cells [47]. Some anticancer agents have been reported to induce autophagic cell death in some malignant cell types [1], [42]. On the contrary, SN09-2 promotes rather direct death signals leading to apoptosis. The differential activation of death mechanisms induced by these antagonists is a likely explanation of the fact that SN09-2 has potent cell death activity compared with Trp-1.

A xenograft model with prostate cancer cells revealed that SN09-2 inhibits tumor growth without any apparent adverse effects to the animal. However, the anti-tumor activity was much lower than that of Trp-1 with respect to tumor volume regression. Because DMSO which is a solvent of SN09-2 for *in vitro* cell treatment may be not recommended for *in vivo* use, we dissolved SN09-2 with DMA, which was previously used for Trp-1. Several days after injection, we observed a solid mass at the injection site, indicating that unresolved reagents were retained at the site. This mass may not stimulate inflammatory responses, since no skin irritation or densely populated cells were detected. Unfortunately, we failed to identify a good solvent for SN09-2 in spite of attempts with many biocompatible carriers. However, if an appropriate solvent for *in vivo* use can be identified, SN09-2 would efficiently inhibit prostate cancer progression.

In summary, we demonstrate that the GnRH-II antagonist SN09-2 exerts a growth inhibitory effect on prostate cancer cells. The accumulation of SN09-2 in the mitochondria of prostate cancer cells may indicate that SN09-2 directly affects mitochondrial dysfunction, leading to the induction of ROS generation, cytochrome c release, and subsequent caspase-3 activation, which are critical steps towards apoptotic cell death. The powerful anti-proliferation activity of SN09-2 and its action mechanism reinforces the idea that GnRH-II antagonists may be developed as weapons to treat prostate cancer.
